# Exploring the Mechanism Responsible for Cellulase Thermostability by Structure-Guided Recombination

**DOI:** 10.1371/journal.pone.0147485

**Published:** 2016-03-17

**Authors:** Chia-Jung Chang, Cheng-Chung Lee, Yueh-Te Chan, Devin L. Trudeau, Mei-Huey Wu, Chih-Hsuan Tsai, Su-May Yu, Tuan-Hua David Ho, Andrew H.-J. Wang, Chwan-Deng Hsiao, Frances H. Arnold, Yu-Chan Chao

**Affiliations:** 1 Institute of Molecular Biology, Academia Sinica, Taipei, Taiwan, ROC; 2 Institute of Biological Chemistry, Academia Sinica, Taipei, Taiwan, ROC; 3 Core Facilities for Protein Structural Analysis, Academia Sinica, Taipei, Taiwan, ROC; 4 Division of Chemistry and Chemical Engineering, California Institute of Technology, Pasadena, California, United States of America; 5 Institute of Biotechnology, National Cheng Kung University, Tainan, Taiwan, ROC; 6 Institute of Plant and Microbial Biology, Academia Sinica, Taipei, Taiwan, ROC; 7 Agricultural Biotechnology Center, National Chung Hsing University, Taichung, Taiwan, ROC; Università degli Studi di Milano, ITALY

## Abstract

Cellulases from *Bacillus* and *Geobacillus* bacteria are potentially useful in the biofuel and animal feed industries. One of the unique characteristics of these enzymes is that they are usually quite thermostable. We previously identified a cellulase, GsCelA, from thermophilic *Geobacillus sp*. 70PC53, which is much more thermostable than its *Bacillus* homolog, BsCel5A. Thus, these two cellulases provide a pair of structures ideal for investigating the mechanism regarding how these cellulases can retain activity at high temperature. In the present study, we applied the SCHEMA non-contiguous recombination algorithm as a novel tool, which assigns protein sequences into blocks for domain swapping in a way that lessens structural disruption, to generate a set of chimeric proteins derived from the recombination of GsCelA and BsCel5A. Analyzing the activity and thermostability of this designed library set, which requires only a limited number of chimeras by SCHEMA calculations, revealed that one of the blocks may contribute to the higher thermostability of GsCelA. When tested against swollen Avicel, the highly thermostable chimeric cellulase C10 containing this block showed significantly higher activity (22%-43%) and higher thermostability compared to the parental enzymes. With further structural determinations and mutagenesis analyses, a 3_10_ helix was identified as being responsible for the improved thermostability of this block. Furthermore, in the presence of ionic calcium and crown ether (CR), the chimeric C10 was found to retain 40% residual activity even after heat treatment at 90°C. Combining crystal structure determinations and structure-guided SCHEMA recombination, we have determined the mechanism responsible for the high thermostability of GsCelA, and generated a novel recombinant enzyme with significantly higher activity.

## Introduction

Cellulases, including endoglucanases (EC3.2.1.4), cellobiohydrolases (EC3.2.1.91) and beta-glucosidases (EC 3.2.1.21), convert cellulosic materials into renewable energy and commodity chemicals [1]. Thermophilic cellulases are desirable in such applications since their activity at higher temperatures could result in shorter hydrolysis times [2], decreased risk of contamination [3], facilitated recovery of volatile products such as ethanol [4], and lower costs for cooling after thermal pretreatment [5, 6]. We have previously isolated and characterized a novel cellulase, GsCelA, from a thermophilic *Geobacillus sp*. 70PC53, which had higher endoglucanase activity than commercial enzymes such as Celluclast 1.5L. In addition, GsCelA was active from 50 to 70°C, and retained 70% activity after 4h at 75°C [7]. However, the molecular basis for thermal tolerance in this enzyme had not been investigated.

Cellulases are usually more stable than general enzymes in functioning at relatively high temperatures. Thermophilic bacteria belonging to the strains *Bacillus*, *Geobacillus*, *Caldibacillus*, *Acidothermus*, *Caldocellum* and *Clostridium* are known to produce thermostable cellulases [8, 9]. *Bacillus* and *Geobacillus* strains are industrial thermophilic bacterial strains widely used in the production of value-added vitamins, enzymes and proteins [8, 9]. The GsCelA enzyme considered in this study belongs to a particular group of *Geobacillus*. The recombinant GsCelA expressed in *E*. *coli* exhibited ten-fold greater specific activity than the commercially available endo-glucanase from *Trichoderma reesei* and uniquely retained its activity after long-term heating and low pH treatments [7]. The amino acid sequence of GsCelA indicates it is a member of the glycoside hydrolase GH5 family of cellulases, but shares only 53.1% similarity with other members in this group [7]. In contrast to its full-length sequence, the catalytic core of GsCelA has 60% homology with that of BsCel5A from *Bacillus subtilis* 168.

BsCel5A, another cellulase belonging to the GH5 enzymes, is the major endoglucanase in *Bacillus*. BsCel5A from different *Bacillus subtilis* strains have been cloned and characterized for their application in biofuel production [10, 11]. BsCel5A is also a thermostable enzyme, though it is not as tolerant at high temperatures as GsCelA, retaining 70% of its optimal activity after incubation at 75°C for 30 minutes or less. A TIM-barrel (α/β)_8_ catalytic domain and a β-sheet cellulose binding module (CBM3) were shown to be present in the cellulase BsCel5A [11].

In contrast to BsCel5A, the structure and mechanism of GsCelA has not been investigated. Therefore, comparisons of GsCelA and BsCel5A provide a unique opportunity for us to explore the mechanism contributing to the better stability of GsCelA at higher temperatures. Here, we have applied SCHEMA structure-guided protein recombination technology to address the mechanism by which GsCelA structure contributes to better thermostability.

The SCHEMA algorithm uses structural information to select boundary locations that minimizes disruption of favorable residue-residue contacts in the resulting chimeras [12]. Non-conserved sequence elements, or sequence ‘blocks’, are then shuffled among homologous proteins (parental proteins) to generate functional chimeras. Because these blocks contribute to chimera stability with a high degree of additivity, stabilized chimeras can be predicted using models built by sampling a small set of chimeras [13]. In addition, the residues that contribute to stabilizing protein structure may also be identified in the process [14–16].

In this study, BsCel5A, a much less thermostable endoglucanase from *Bacillus subtilis* 168 [17, 18], which shares 60% sequence homology with GsCelA, was selected as the second parental protein for SCHEMA recombination. As a result, a highly thermostable chimeric cellulase C10 with increased activity was developed. Through crystal structure determination of GsCelA and C10, we uncovered a 3_10_ helix structure that is responsible for the higher thermostability of the enzyme. Moreover, we discovered that addition of crown ether (CR), a crystallization additive and a modulator of protein surface behavior [19], could further improve the thermostability of this cellulase. These newly-discovered structural features have contributed to our knowledge of the mechanisms for endowing better thermostability and activity amongst bacterial GH5 cellulases. These mechanisms may also be applied to improving the thermostability and/or activity of other proteins or enzymes.

## Materials and Methods

### Protein expression and purification of chimeras and mutants

Thirteen chimeric GH5 genes were optimized for expression in *E*. *coli*, and the gene sequences were synthesized by GenScript, U.S.A. The PCR fragments and synthesized gene sequences were cloned into CloneJET^™^ PCR cloning vector (Thermo Scientific) and subsequently subcloned into the pET21b expression vector (Novagen) using the restriction sites *Nde*I and *Xho*I. Site-directed mutagenesis and short-fragment substitutions were performed using the QuikChange Lightning Site-directed Mutagenesis Kit (Agilent Technologies) and In-Fusion^™^ HD Cloning system (Clonetech). The proteins were expressed in BL21(DE3) *p*Lyss at 37°C for 4–6 hrs under the induction of 0.5 mM IPTG (isopropyl *β*-D-thiogalactopyranoside). Harvested cells were resuspended in binding buffer (50 mM sodium phosphate, pH 8, 300 mM NaCl) and disrupted by sonication. Lysates were centrifuged at 10000 x *g* for 60 min and the supernatants were loaded onto nickel-charged 1 ml HiTrap FF crude columns (GE Healthcare) with a flow rate of 0.5 ml/min. The proteins were eluted by 150–300 mM imidazole. All chromatographic steps were performed using an AKTA FPLC system (GE Healthcare). The purified protein samples were confirmed by SDS-PAGE under denaturing conditions and their concentrations were determined by Bradford assay.

### Noncontiguous SCHEMA recombination

SCHEMA is a structure-guided computational approach to creating chimeric proteins that retain proper folding and functionality, but explore other properties linked to sequence, such as stability. SCHEMA algorithms identify sites for recombining homologous proteins that minimize structural disruption by maximizing the retention of parental residue-residue contacts in their folded structures [20]. Noncontiguous recombination identifies blocks of sequence that are contiguous in the 3-D structure, but are not necessarily contiguous in the primary sequence. Contacts (residues that are < 4.5 Å apart) are identified from one or more of the crystal structures, and the SCHEMA energy E for a given chimera is calculated by counting the number of residue–residue contacts that are disrupted by recombination. Partition sites of the aligned homologous proteins are chosen to minimize the average of SCHEMA energy <E> of all possible chimeras made by recombining those sequence fragments.

In this study, noncontiguous SCHEMA recombination was designed as previously described [12]. The SCHEMA algorithms uses sequence alignment and structure data to create a SCHEMA contact map for proper chimera design. In the generated structures, the algorithm was set to consider any two amino acids in contact if any atoms, excluding hydrogen, are within a distance of 4.5Å from the residues. A SCHEMA contact map was first generated for each parent. During recombination, the contacts that are not conserved among the parental proteins were considered broken, and so a final ‘average’ contact map could be built by weighting the retention of each parental contact (0.5 for a single parent, 1 for both parents). The SCHEMA contact map can be abstracted as a graph in which every node represents a non-conserved residue, and is linked by the edges representing the average weighted SCHEMA contacts between two residues. The problem of finding crossover locations that minimize the SCHEMA contact numbers to yield the low-disruption chimeras can therefore be reformulated as the problem of minimizing the edges during graph partitioning, which was solved with the hMETIS graph partitioning suite [21, 22].

Here, the amino acid sequence alignment of the parental enzymes BsCel5A [11] and GsCelA [7] was created using PROMALS3D.24. Crystal structures 3PZT [11] and 4XZB were used to create the BsCel5A and GsCelA SCHEMA contact maps, respectively. As the catalytic cores of BsCel5A and GsCelA share 58% sequence identity, an eight-block chimera design was selected with an average <E> as 16.25 and average <m> as 47 compared to the closest parent.

### Measurement of thermostability

In the *T*_50_ thermostability assay, 1 μg of purified cellulase was mixed with 50 mM sodium acetate, pH 5.0, in a final reaction volume of 300 μl. The tubes were incubated in a gradient thermocycler for 10 min with or without 0.05 mM additional calcium ion (Ca^2+^) and/or 6.25x10^-3^ mM crown ether (18-crown-6, CR [19]) at a range of temperatures, and then cooled to 4°C. To each tube, 3 μg of phosphoric acid swollen cellulose (PASC) [23] was added, and the heat-treated cellulases were allowed to react at their optimal temperatures for 6 hrs in a thermal cycler. The amount of reducing sugars was measured and quantified by the DNS method [24]. The parameter *T*_50_ is the temperature at which an enzyme loses 50% of its optimal activity after a 10-min heat treatment [13].

The *T*_A50_ thermostability assay was performed in an 8-well PCR strip, into which 1 μg purified cellulase was mixed with 50 mM sodium acetate, pH 5.0, and 3 μg PASC in a final reaction volume of 300 μL. The tubes were incubated at a range of temperatures for 6 hrs in a gradient thermocycler and then cooled to 4°C. The reducing sugars were measured and quantified by the DNS method [24]. The parameter *T*_A50_ is the temperature at which a cellulase exhibits 50% of its optimal activity in a 6-hr hydrolysis assay [13].

### pH tolerance measurement

To test for pH tolerance, the purified enzymes were incubated in 50 mM buffer at different pH values between 2.0 and 10.0 for 12 hrs. Residual activities were then measured with 1% PASC in 50 mM sodium acetate buffer, pH 5.0, at optimal temperatures using the DNS method [24].

### Long-term cellulase activity assay

All cellulase activity measurements were conducted in 50 mM sodium acetate buffer, pH 5.0. For PASC (1% w/v) hydrolysis assays, 300 μl of reaction volume containing 1 μg of purified parental or chimeric GH5 cellulases and 0.5 μg Novo-188 (Novozyme) were constituted, and then the reaction was allowed to proceed at 50°C for 6–60 hrs. After hydrolysis, the reaction supernatants were collected, and the reducing sugar concentration was measured by the DNS method [24].

### Circular dichroism measurement

Far-UV CD spectra (190–260 nm) were recorded on an AVIV model 202 CD spectrometer using a 1-mm quartz cuvette. Proteins were used at a concentration of 10 μM in 50 mM sodium phosphate buffer, pH 8. Data collection parameters were set to a scan rate of 50 nm/min, response time of 4 s, sensitivity of 100 mdeg, accumulation of 10, heating rate of 1°C/min and 60-s delay time for spectrum collection. Results were expressed as mean residue ellipticity (deg · cm2 ·dmol^−1^ · residue^−1^). All thermal unfolding experiments were monitored at 222 nm.

### Crystallization and data collection

The crystals of GsCelA P1 and C10 were grown by mixing 1 μl protein with 1 μL reservoir solution using the sitting-drop vapor diffusion method at 18°C. P1 crystals were obtained in a reservoir solution of 16% (*w/v*) PEG 4000, 0.2 M imidazole malate, pH 6.0. C10 crystals were grown in a reservoir solution of 25 mM 18-crown-6, 30% (*w/v*) PEG 4000, 0.1 M HEPES sodium salt at pH 7.4, and 0.2 M calcium chloride. Both crystals were flash-cooled with 22% glycerol (*v/v*) as a cryo-protectant. Diffraction data for P1 crystals were collected at cryogenic temperatures at a wavelength of 1.5418 Å using a Rigaku FR-E+ SuperBright generator equipped with an R-AXIS HTC image-plate detector. Data for the C10 crystals were collected at a wavelength of 1.00 Å on beam line BL12B2 of the Spring-8 synchrotron in Japan using a Quantum-210 CCD detector. All diffraction data were processed and scaled using the HKL2000 program [25].

### Structural determination and refinement

Both crystal structures (P1 and C10) were determined by molecular replacement using the MOLREP program of the CCP4 program suite, using the crystal structure of endo-1, 4-beta-glucanase (PDB: 3PZT) from *Bacillus subtilis* [11] as a search model. P1 and C10 crystals belong to space groups *P*2_1_2_1_2_1_ and *C*222_1_, respectively. Throughout the refinement, 5% of randomly-selected data were set aside for cross-validation with *R*_free_ values. Manual modifications of the models were performed using the program Coot [26]. Difference Fourier (Fo-Fc) maps were calculated to locate the solvent molecules. Both crystal structures were refined using Refmac5 [27]. Data collection and final model statistics are shown in S1 Table. The molecular figures were produced using UCSF Chimera [28]. The atomic coordinates and structural factors of P1 and C10 have been deposited in the Protein Data Bank with accession codes 4XZB and 4XZW, respectively. The molecular figures were produced using *PyMOL* (http://www.pymol.org) and UCSF Chimera [28].

## Results

### Library design of noncontiguous recombination between endo-β-1,4-glucanases from the thermophiles *Geobacillus* and *Bacillus*

**Fig 1:**
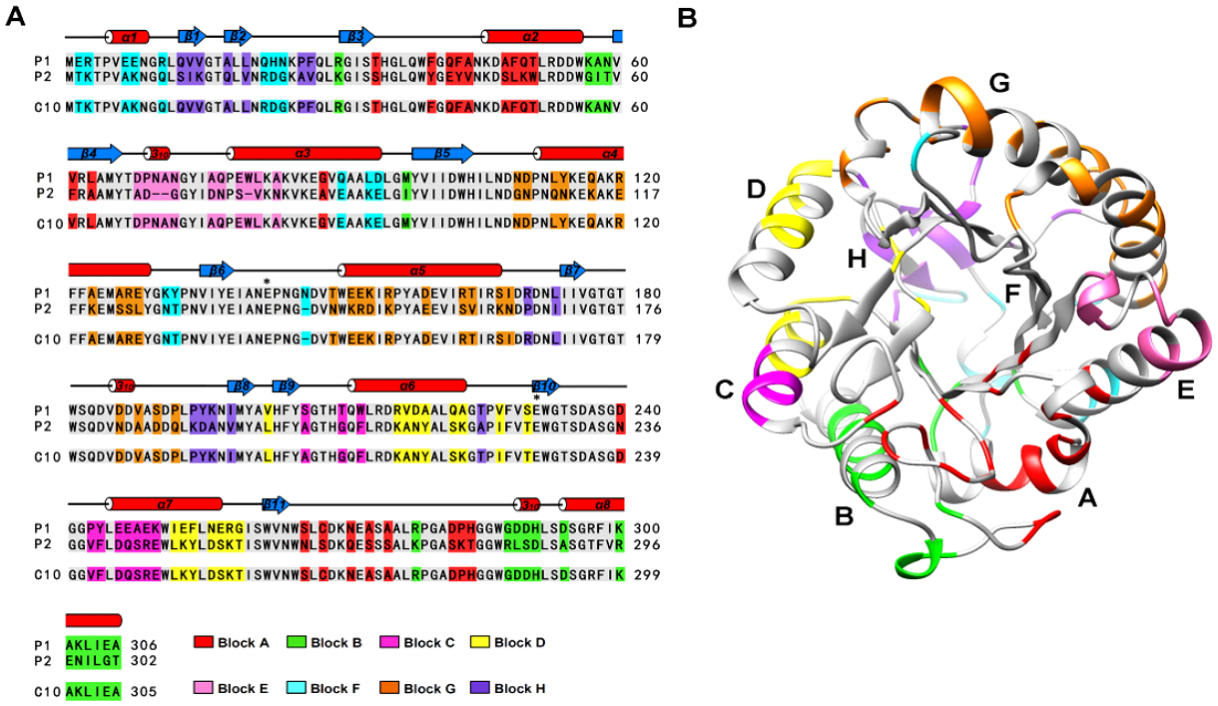
Sequence alignment and noncontiguous blocks of the cellulases GsCelA and BsCelA. (A) The C10 chimera sequence is aligned with P1 and P2 parental sequences. Residues in the α-helices and β-strands are indicated by red cylinders and blue arrows, respectively. P1 is the catalytic core of thermostable GsCelA from *Geobacillus* sp. 70PC53, and P2 is the catalytic core of *Bacillus subtilis* 168 BsCel5A. The asterisks indicate catalytic acids and bases. (B) Noncontiguous blocks in the designed GH5 chimera. The eight noncontiguous blocks indicated by colored regions were calculated using the SCHEMA program at <E> = 16.25 and <m> = 47. Amino acids that are the same in both parent enzymes are shown in grey.

Two bacterial GH5-family enzymes were chosen as parents for noncontiguous SCHEMA recombination: BsCel5A [11] from the thermophile *Bacillus subtilis* 168, and GsCelA from the highly thermophilic *Geobacillus* sp.53 [7] (Fig 1A). The crystal structure of the *Geobacillus* sp.53 GsCelA catalytic domain (GsCelA P1) was first determined at 1.62 Å resolution. GsCelA P1 crystallized in the *P*2_1_2_1_2_1_ space group, with one protein molecule per asymmetric unit (S1 Table). As shown in Fig 1B, GsCelA P1 adopts a TIM-barrel fold, similar to that of the BsCel5A catalytic domain. The 60% sequence similarity and 58% identity in the catalytic domain with closed structure superposition suggested that these two enzymes could be recombined to yield functional catalytic domain chimeras.

We designed a noncontiguous SCHEMA recombination library of GH5-family catalytic domains from GsCelA (P1) and BsCel5A (P2) that yielded an <E> of 16.25 and an average of 47 mutations (<m>) from the closest parent. The individual structural elements, or blocks, for this design are shown in Fig 1A. The designed chimeras were assembled from 16 gene fragments of two parents, with each containing approximately 16 non-conserved residues, representing the eight blocks from each of the parents.

### Expression and functional assessment of chimeric GH5 cellulases

From a library of 2^8^ = 256 chimeric sequences, we selected a subset of eight chimeras for construction and characterization (Table 1, chimeras C1 to C8). These chimeras were chosen to maximize mutual interaction among the sequences (m values) and to minimize SCHEMA disruptions (E values), as has previously been described for library designs of chimeric argininases and cellobiohydrolases [29, 30]. Therefore, chimeras with higher m and lower E values were chosen. The parents (P1 and P2) and all eight chosen chimeras (C1–C8) were expressed and purified from *E*. *coli*.

**Table 1:** Informative subsets of parental, chimeric, and designed GH5 cellulases.

Parents (P)	ABCD EFG H	SCHEMA E	*m*	*T*_A50_ (°C)^1^	*T*_50_ (°C)^2^	A_re_^3^*
	P1 1 1 1 1 1 1 1 1	0	0	80 ± 0.9	79 ± 0.4	1.17 ± 0.06
	P2 2 2 2 2 2 2 2 2	0	0	64 ± 0.6	72 ± 0.3	1.00
Identified active chimeras (C)	C1 2 2 2 1 1 1 1 1	10	51	62 ± 0.5	69 ± 0.5	1.21 ± 0.07
	C2 2 2 1 1 2 1 1 1	7	52	50 ± 0.6	67 ± 0.4	0.94 ± 0.05
	C3 1 1 1 1 1 2 2 2	9	52	64 ± 0.7	66 ± 1.0	0.92 ± 0.06
	C4 2 2 1 1 1 2 1 1	14	55	64 ± 0.6	68 ± 0.9	0.84 ± 0.05
	C5 2 1 1 1 1 1 1 1	8	21	64 ± 0.9	77 ± 0.8	1.42 ± 0.07
	C6 1 1 1 1 1 1 2 1	9	24	60 ± 1.0	68 ± 0.6	1.34 ± 0.08
	C7 2 2 1 1 1 1 1 1	6	41	58 ± 1.1	64 ± 1.2	1.35 ± 0.06
	C8 2 2 2 2 1 1 1 1	8	63	68 ± 0.9	80 ± 1.4	1.23 ± 0.06
Predicted active and stable chimeras (C)	C9 1 1 2 2 2 2 1 1	22	51	67 ± 1.2	76 ± 1.1	1.18 ± 0.04
	C10 1 1 2 2 1 2 1 1	21	40	76 ± 1.1	83 ± 0.8	1.43 ± 0.09
	C11 1 1 2 2 1 1 1 1	12	26	70 ± 0.6	81 ± 0.9	1.40 ± 0.07
	C12 2 1 2 2 2 2 2 2	11	20	70 ± 0.4	74 ± 0.5	1.37 ± 0.06
	C13 2 2 2 2 1 2 2 2	0	11	71 ± 1.0	73 ± 0.6	1.39 ± 0.04

^1^ T_A50_ is the temperature at which an enzyme has 50% of its optimal activity.

^2^ T_50_ is the temperature at which an enzyme loses 50% of its activity after 10 min pre-incubation.

^3^ A_re_ is the cellulase’s specific activity at its optimal temperature measured in a 10 min assay with 1 μg purified enzyme and 1% PASC. The values are normalized relative to the specific activity of BsCel5A catalytic core.

* All of these enzymes are assayed at their optimal temperature at 60°C except that the optimal temperatures of C2 and C3 are 55°C; and P2, C5, C6, C7, and C12 are 50°C.

We then evaluated the activity and thermostability of the eight sample chimeras and their parents based on two measurement parameters: *T*_50_ and *T*_A50_. In short, *T*_50_ describes the tolerance of an enzyme to thermal stress, and *T*_A50_ describes its ability to function at elevated temperature. The *T*_50_ of six chimeras (C1, C2, C3, C4, C6 and C7 in Table 1) suggested they were less stable than the parent species. Two chimeras (C5 and C8) were more stable than one parent, but only the *T*_50_ of C8 was higher than both of the parent, including the most stable parent, P1.

### Model prediction of thermostability and thermoactivity

**Fig 2:**
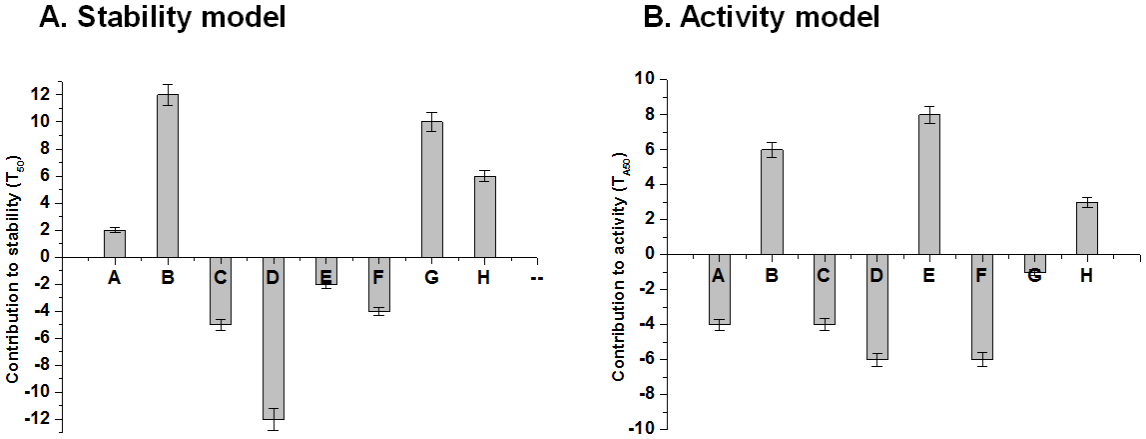
Modeling of thermostability and thermoactivity of GH5 chimeras. (A) Stabilizing or destabilizing effects of 8 noncontiguous blocks, relative to P2 (BsCel5A) for the *T*_50_ model. Blocks A, B, G and H of P1 are predicted to be stabilizing, but replacement of C, D, E and F had negative effects on thermostability. (B) Stabilizing or destabilizing effects of 8 noncontiguous blocks, relative to P2 for the *T*_A50_ model. Blocks B, E and H of P1 are predicted to be stabilizing.

Several reports have shown that the SCHEMA recombined contiguous blocks of sequences contribute additively to chimera stabilities and that these stabilities are predictable with simple additive block models trained on a sample set of a library [31]. Thus, our small but informative 8-chimera GH5 endoglucanase library could be used to construct a linear model assuming that individual blocks of the structure contribute additively to the stabilities of recombined enzymes. We constructed predictive models of *T*_50_ and *T*_A50_ based on the sequences of our eight functional chimeras and two parental cellulases by linear regression. The *T*_50_ model predicts the stabilities of the library sample (r^2^ = 0.83) and provides the predicted contributions of each structural block to *T*_50_ (Fig 2A). Similarly, we plotted a model that fits the *T*_A50_ stability data (r^2^ = 0.88). The predicted block contributions to *T*_A50_ are shown in Fig 2B.

According to the two thermostability models and their block predictions, we synthesized an additional five chimeras (C9 to C13, Table 1) to better identify key elements contributing to thermostability. The C9 and C10 chimeras were predicted to be more stable than the parents according to the *T*_50_ model. Of these, C10 was significantly more stable: its *T*_50_ was enhanced by 4°C compared with P1 and 9°C compared with P2. The C9, C10, and C11 chimeras were used to assess the contributions of blocks F and E (Table 1). Following swapping between chimeras, we found that block E of P1 and block F of P2 enhanced the thermostability of C10.

### Characterization of the most active and stable chimera, C10

**Fig 3:**
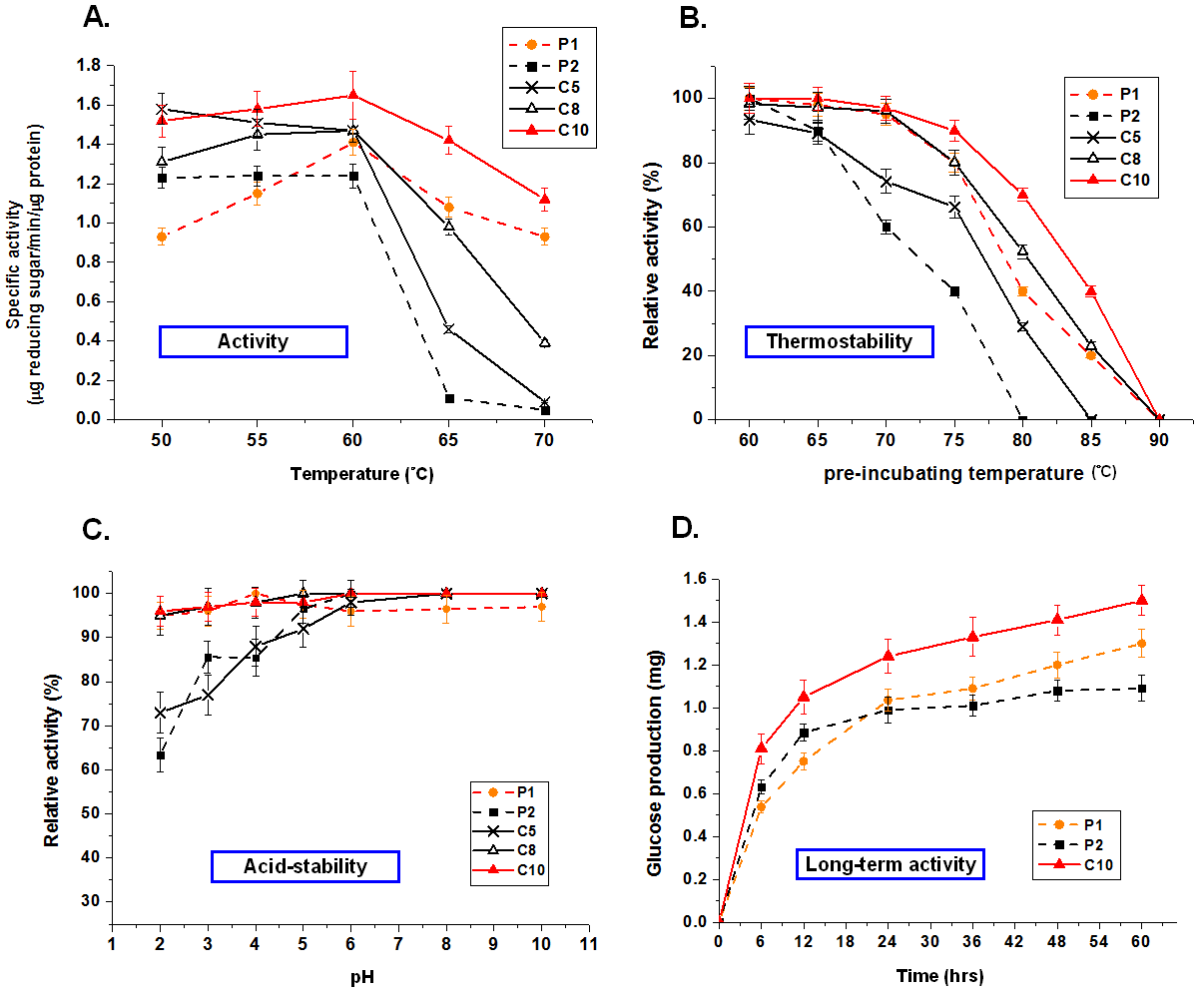
Characterization of GH5 chimeras. (A) Activity profiles of GH5 parents and chimeras at high temperatures. (B) Residual activities (thermostability) of GH5 parents and chimeras after heat-treatment. One microgram of purified cellulases was pre-incubated in 50 mM sodium acetate buffer (pH 5.0) at different temperatures for 10 min. The residual enzyme activities were then determined on 1% PASC at pH 5.0 at their optimal temperature for 6 hrs. Relative activities were compared with the optimal activity of each enzyme without a pre-incubating treatment at 60°C. Some of the enzymes, e.g. C5, do not have the initial point (60°C) of 100% because their optimal activities are not at this reaction temperature and therefore have a slightly lower staring points in the analyses with 60°C pre-incubation temperature. (C) Acid stability of the parents and chosen chimeras. One microgram of purified cellulases was pre-incubated in 50 mM buffer with variable pH for 12 hrs. The residual enzyme activity was determined. Relative activities were compared with the optimal activity of each enzyme without a pre-incubating treatment at pH 5. (D) Long-term activity of the most stable chimera C10 compared with its parents in PASC hydrolysis. One microgram of purified cellulases plus 0.5 μg Novo-188 (β-glucosidase, Novozyme) was added into 1% PASC at 50°C, pH 5.0 for 6, 12, 24, 36, 48, and 60 hrs.

The C10 chimera was predicted and synthesized based on the experimental thermostability results of the eight sampled active chimeras. Our results showed that C10 was more catalytically active and more stable than the parent GsCelA (P1), which was already more thermostable than P2. In addition to C10, we also chose two other chimeras (C5 and C8), both of which are less stable than P1 but more stable than P2, to characterize their optimal temperature, thermostability, pH tolerance, and long-term activity (Fig 3). C10 exhibited both broader and higher thermoactivity than P1 in the range of 50°C to 70°C (Fig 3A), with C10 retaining 70% residual activity after 10 min of 80°C heat-treatment while P1 only retained 40% residual activity (Fig 3B). Interestingly, similar to P1, stable chimeras C8 and C10 had higher acid-stability than C5 and P2 (Fig 3C). In long-term hydrolysis of amorphous cellulose, the most stable chimera, C10, maintained activity longer than the GH5 parents (Fig 3D).

### A new 3_10_ helix contributes to thermostability of bacterial GH5 cellulases

**Fig 4:**
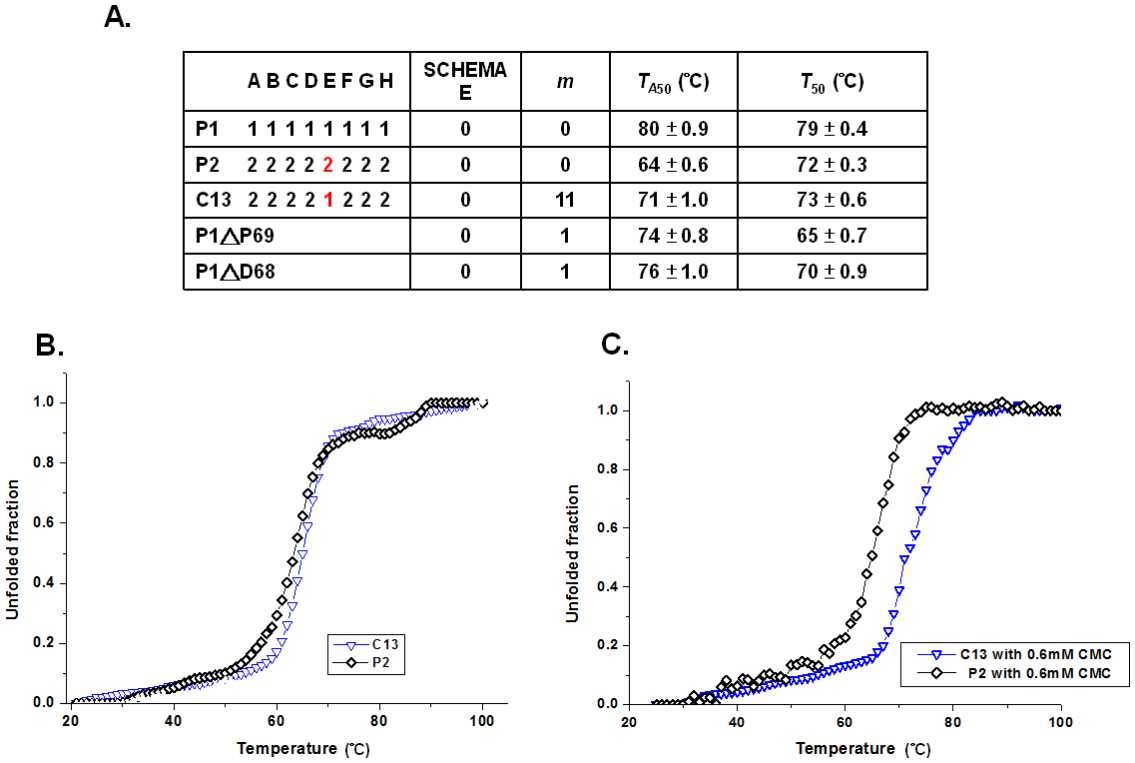
Profile of SCHEMA block E of GsCelA. (A) Thermostabilities of parent enzymes, chimeras and mutants are highlighted for block E. (B) Thermal denaturation curves of P2 (without E loop) and C13 (P2 with additional E loop). (C) Thermal denaturation curves of P2 and C13 in the presence of 0.6 mM CMC (1% w/v).

Since block E of GsCelA had a negative value in the *T*_50_ model but a positive value in the *T*_A50_ model (Fig 2), we studied the contribution of block E to overall thermostability in the protein GsCelA. Block E of GsCelA exhibits a 3_10_ helical structure different from the corresponding sequence in BsCel5A (Fig 1B), and it is located in the catalytic face of the TIM-barrel. As shown in Fig 4A, adding block E of P1 to P2 (see chimera C13) resulted in an increase by 7°C of *T*_A50_ but did not significantly change *T*_50_, compared to P2. In order to further analyze the 3_10_ helical structure of block E, the single residue mutants P1ΔP69 or P1ΔD68, in which P69 or D68 of GsCelA was deleted, respectively, exhibited a significant drop in both *T*_A50_ and *T*_50_ compared with wild-type GsCelA (P1, Fig 4A).

**Fig 5:**
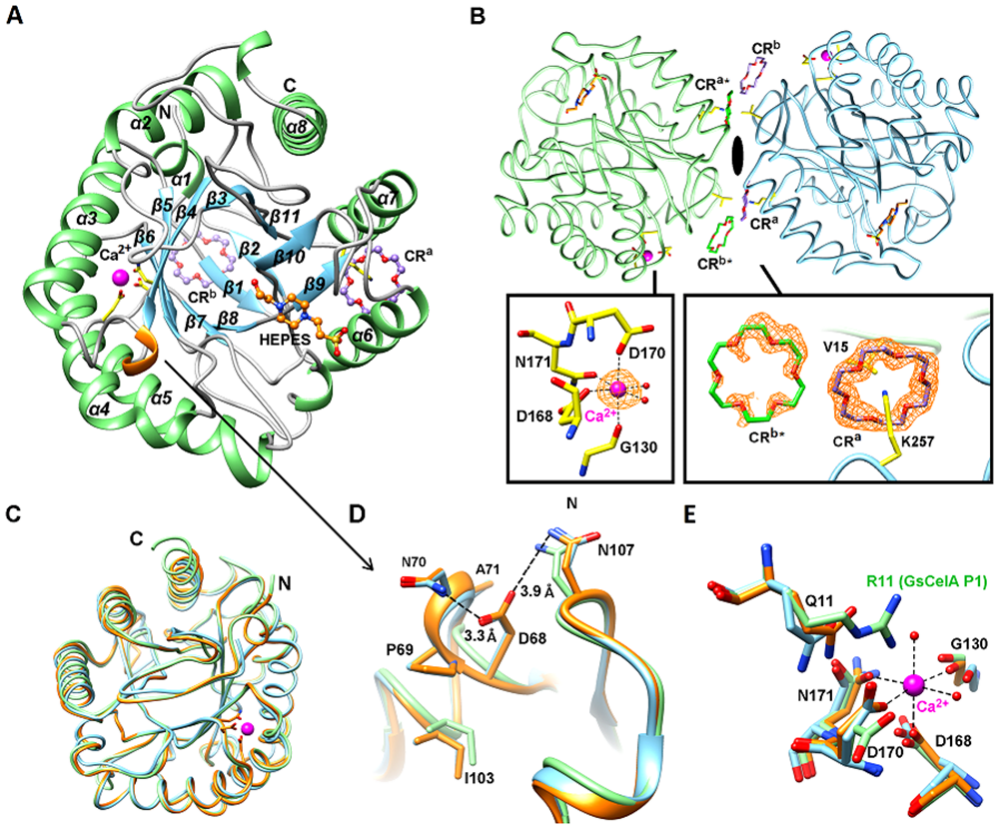
Crystal structure of chimera C10/crown ether complex. (A) The overall structure of C10 with HEPES, two CR molecules, and a calcium ion (ball-and-stick models). (B) Two C10 monomers (light green and light blue) from different unit cells are in contact with 2-fold symmetry. Two CR molecules interacting across different unit cells, CR^a^ and CR^b+^, are shown as purple and green carbon atoms, respectively. Fo-Fc omit maps (orange) were calculated for the Ca^2+^ and CR molecules at 2 σ level. The calcium ion is bound to two water molecules, the backbone oxygen of G130 and the three carboxylate side chains of D168, D170 and N171, with an octahedral configuration. A CR molecule (CR^a^) is bound to K257 and V15 from two different C10 monomers. The second CR molecule (CR^b^) has a hydrophobic interaction with CR^a^. (C) Comparison of GsCelA P1, BsCe5lA P2 (PDB: 3PZT) and chimera C10 structures. The catalytic domains are superimposed and colored in green, blue and orange, respectively. The N and C termini are also indicated. (D) β4α3 loop region comparison. GsCelA P1, BsCe5lA P2 and chimera C10 are colored as before. The D68 side chain atoms are hydrogen bonded to N70 and N107, while P69 has a hydrophobic interaction with I103. The chimera C10 protein residues are shown as stick models and carbon atoms are colored orange. (E) The Ca^2+^ binding sites in GsCelA P1, BsCe5lA P2 and chimera C10 are superimposed. The carbon atoms of GsCelA P1, BsCe5lA P2 and chimera C10 are colored in light green, light blue and orange, respectively. The Ca^2+^ atom from GsCelA P1 is depicted as a light magenta sphere.

As shown in Fig 5A and 5D, the determined C10 structure exhibited side-chain bonding of D68, forming a 3_10_ helix with amino acids P69, N70 and A71. The 3_10_ helix is inserted between strand β4 and helix α3, and is packed into the concave cavity between two loops, β4α3 loop and β6α4 loop. These two loops are stabilized by the hydrogen bond formed between the side chains of D68 and N70. Furthermore, the hydrophobic side chain of P69 undergoes a nonpolar interaction with residue I103, further contributing to the stabilization of the β4α3 loop. We speculate that the additional 3_10_ helix may contribute either increased protein rigidity or stabilized interactions of enzyme-substrate in the transition state. To confirm that this was the cause of the enhanced thermoactivity, we measured the thermal denaturation of these parental and mutated proteins in the presence or absence of substrate (Fig 4B and 4C). Interestingly, when P2 and C13 were incubated with the soluble substrate carboxymethylcellulose (CMC), the C13 chimera exhibited a higher melting temperature than P2 (Fig 4C). The addition of CMC may mimic actual interaction between enzyme and substrate in the thermal unfolding process, and C13 is further stabilized by subtract binding.

### Addition of crown ether enhances thermostability

As reported previously, crown ether (CR) is a powerful crystallization additive and can be used to modulate protein surface behaviors [19]. It has also been found that CR interacts with lysines and protein hydrophobic patches. Therefore, in an attempt to further enhance the thermostability of the C10 chimera, we used CR as an additive for crystallization and in the thermostability assay.

**Fig 6:**
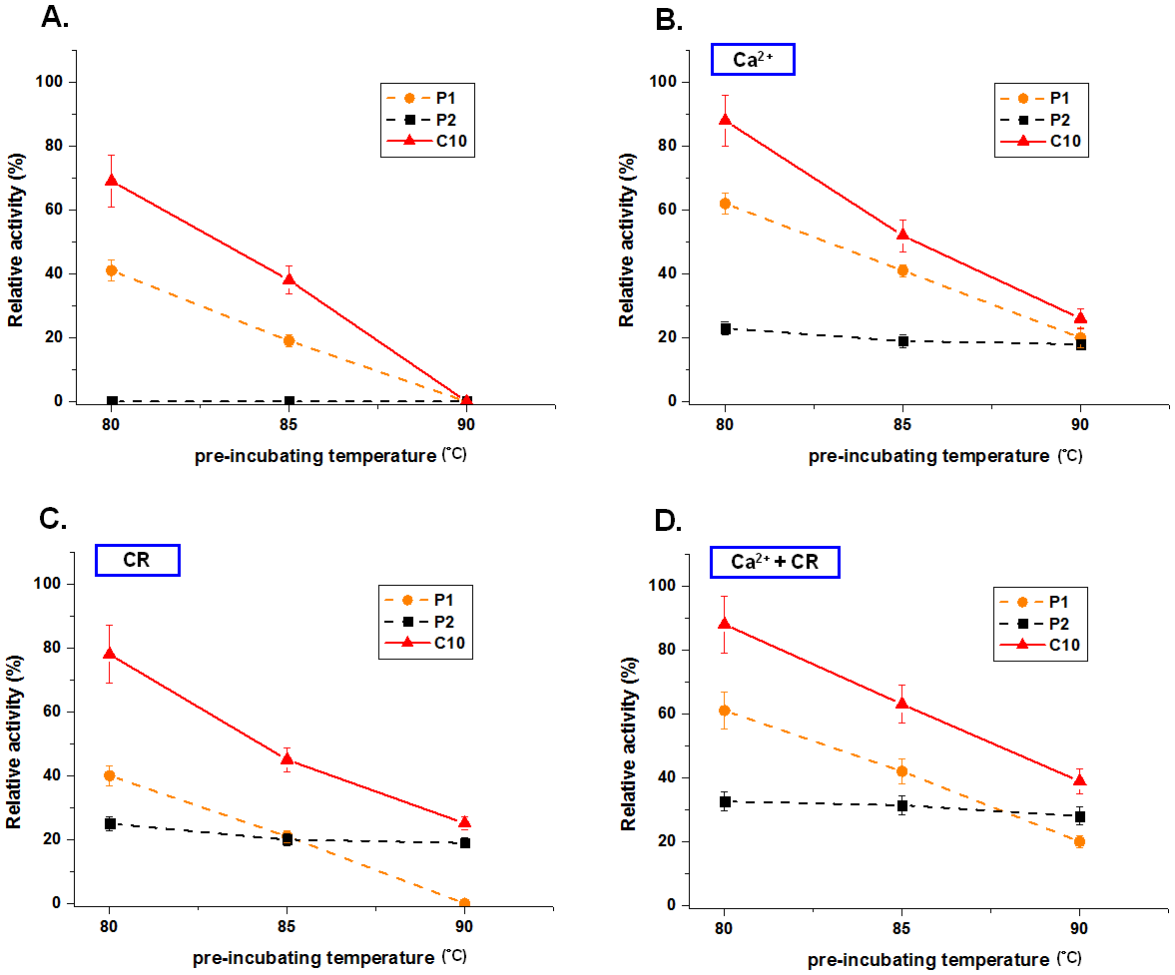
The thermostability of C10 is enhanced by addition of Ca^2+^ and CR. Thermotolerance of the C10 chimera and parental enzymes in the absence of Ca^2+^ (A), presence of Ca^2+^ (B), presence of CR (C), and presence of both Ca^2+^ and CR (D). Purified cellulases (1μg) were pre-incubated in 50 mM sodium acetate buffer (pH 5.0) at different temperatures for 10 min with or without additional Ca^2+^ 0.05 mM or/and CR 6.25×10^−3^ mM. The residual enzyme activity was then determined on 1% PASC at pH 5.0 at their optimal temperature for 6 hrs. The 100% value of relative activity refers to the optimal activity of each enzyme without thermal treatment.

The complex crystals of chimera C10/CR belong to the *C*222_1_ space group with one protein molecule per asymmetric unit, plus HEPES, a calcium ion (Ca^2+^), and two CR-bound molecules. As shown in Fig 5, the structure of C10 adopts the same classical TIM barrel fold as the parental structures of GsCelA core (P1) and BsCel5A (P2) (PDB: 3PZT). Chimera C10 superimposes well on P1 and P2 (Fig 5C), yielding rmsd values of 0.486 Å for 300 atoms and 0.581 Å for 282 atoms, respectively. Interestingly, the chimera C10/CR complex structure revealed a dimeric packing mode with two CR molecules. The first CR adopts a KC-crown binding mode [19], interacting with K257 and V15 from two different chimera C10 monomers, while the second CR is in hydrophobic contact with the first CR molecule (Fig 5B). With CR molecules mediating interactions between two C10 chimera molecules, the CR-binding motif was created by the combination of V15 from block H of P1 and K257 from block D of P2. In thermostability assays with CR, P2 and C10 retained 20% residual activity after heat treatment at 90°C (Fig 6C), but the parental GsCelA exhibited no activity (Fig 6A). These data indicate that K257 on parental BsCel5A plays an important role in the interaction with CR for thermostability improvements.

In addition, a Ca^2+^ ion-binding site was identified (Fig 5B). The Ca^2+^ ion is bound to the backbone oxygen of G130 and to the side chains of D168, D170 and N171 with an average distance of 2.3 Å. Two water molecules were also coordinated with distances of 2.3 Å. The Ca^2+^ ion-binding site is similar to the manganese ion (Mn^2+^)-binding site of the BsCel5A catalytic core (P2) (Fig 5E) [11], and both Ca^2+^ and Mn^2+^ ions coordinated to the protein surface with the same octahedral configuration. The presence of the Ca^2+^ ion enhanced the thermostabilities of P1, P2, and C10 (Fig 6A and 6B). When a Ca^2+^ ion and CR were added, C10 retained 40% residual activity even after heat treatment at 90°C, whereas the parental GsCelA exhibited only 20% residual activity (Fig 6D).

## Discussion

To determine the mechanism for thermostability of GsCelA cellulase from *Geobacillus* and to enhance stability improvements for bacterial GH5-family endoglucanases, we first determined the crystal structure of thermostable GsCelA and then used SCHEMA structure-guided protein recombination technology to expand the stability profiles of the GH5 enzymes using its less thermostable homolog, BsCel5A. This information enabled us to efficiently generate a more stable and active chimera, C10. Chimera C10 is more thermostable and acid-stable than its parental GH5 cellulases (Fig 3) and can maintain its higher activity for a longer time, i.e. over 60 hrs at 50°C (Fig 3D). The C10 chimera has the potential to act as a supplement or as part of a thermostable cellulase cocktail [32, 33], or to be used in biomaterial conversion by *Bacillus* transformants [34], as it is similar to both the *Bacillus* and *Geobacillus* parental enzymes.

The determined crystal structures of GsCelA have enabled us to explore the structural mechanism of the improved thermostability. The SCHEMA block E displays a 3_10_ helix conformation that stabilizes the substrate binding loop and by which it can increase the range of cellulase activity at higher reaction temperatures (Fig 3A) rather than retaining residual activity after heat-treatment (Fig 3B). The deletion mutants (P69 or D68) on block E (Fig 4A) also caused GsCelA to become unstable as the hydrogen bonds were broken.

Other than utilizing the SCHEMA protein engineering method, our crystallographic investigation of C10/CR prompted us to adopt the novel approach of supplementing the C10 chimera with “crown ether”, thereby further enhancing its stability (Fig 5). A previous report indicated that crown ethers can modulate the protein surface by interacting with surface lysine residues [19]. The tolerance of the C10 chimera to extreme heat can be enhanced (Fig 6A and 6C) by CR because lysine (K257, Fig 5) resides on the surface of P2 (SCHEMA block D, Fig 1A) instead of arginine as found in GsCelA (P1). Furthermore, we added Ca^2+^ ions when crowning C10, and there is a degree of additivity between the metal ion and the crown ether (Fig 6A and 6D) located on different sites of the C10 surface (Fig 5B).

**Fig 7:**
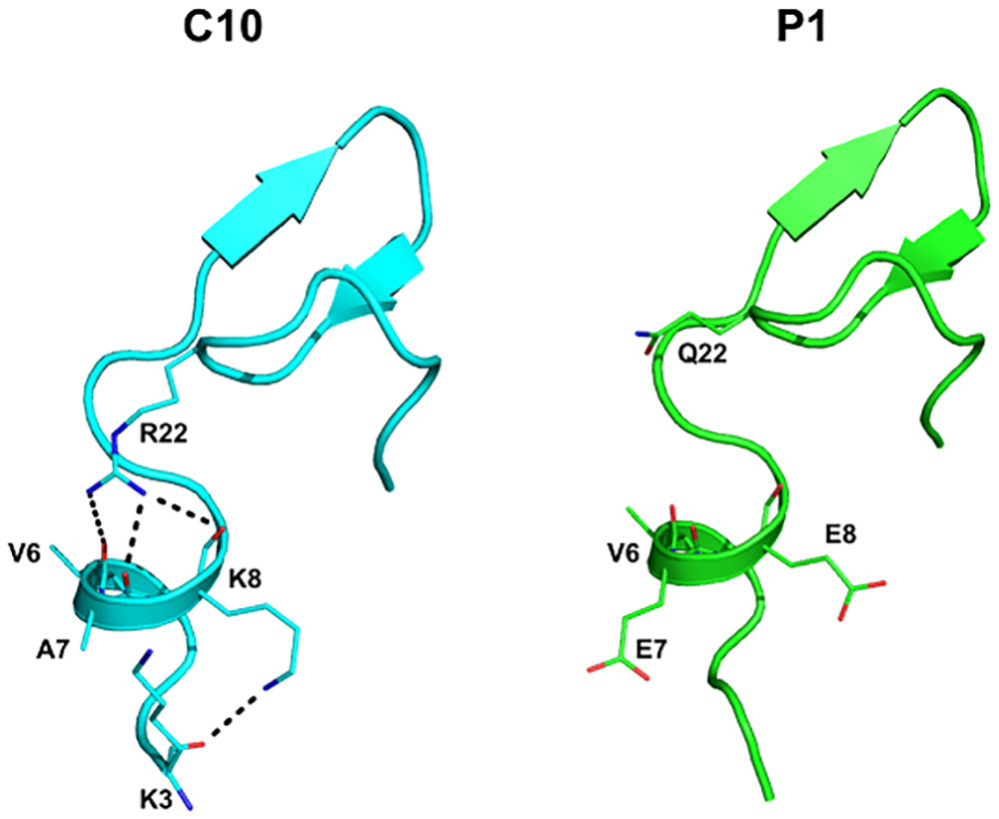
Structural comparison of C10 and P1 at N-termini. C10 undergoes additional interactions through R22 and K8. The nitrogen, oxygen and carbon atoms of C10 are colored blue, red and cyan, respectively, whereas P1 is illustrated with the carbon, oxygen and nitrogen atoms in green, red and blue, respectively. Grey dashes represent hydrogen bonds.

Based on the thermostability models established in this study, we generated a highly stable and active chimera, C10. Superposition of C10 and P1 shows additional interactions occur in block F. R22 in the β2β3 loop of C10 interacts with the backbone oxygen molecules of helix α1 (residues 6–8), and the side chain of K8 forms a hydrogen bond with the backbone oxygen of K3 (Fig 7). These interactions likely stabilize the N-terminal region. Interestingly, the eight residues of the N-terminal region are flexible in P2. After SCHEMA recombination, helix α1 of C10 becomes rigid and new interactions further stabilize this region. This observation is consistent with increased thermostability of C10 compared to C11. In summary, we have identified a novel, stabilizing loop of GsCelA and present a new strategy to maintain high enzymatic activity as well as enhance protein thermostability by additives through SCHEMA engineering.

## Supporting Information

S1 Table Data collection and refinement statistics. (DOCX)

## References

[1] Steen EJ, Kang Y, Bokinsky G, Hu Z, Schirmer A, McClure A, et al Microbial production of fatty-acid-derived fuels and chemicals from plant biomass. Nature. 2010;463(7280):559–62. Epub 2010/01/30. 10.1038/nature08721 nature08721 [pii]. . 20111002

[2] Viikari L, Alapuranen M, Puranen T, Vehmaanpera J, Siika-Aho M. Thermostable enzymes in lignocellulose hydrolysis. Adv Biochem Eng Biotechnol. 2007;108:121–45. 10.1007/10_2007_065 . 17589813

[3] Abdel-Banat BM, Hoshida H, Ano A, Nonklang S, Akada R. High-temperature fermentation: how can processes for ethanol production at high temperatures become superior to the traditional process using mesophilic yeast? Appl Microbiol Biotechnol. 2010;85(4):861–7. Epub 2009/10/13. 10.1007/s00253-009-2248-5 . 19820925

[4] Taylor MP, Eley KL, Martin S, Tuffin MI, Burton SG, Cowan DA. Thermophilic ethanologenesis: future prospects for second-generation bioethanol production. Trends Biotechnol. 2009;27(7):398–405. 10.1016/j.tibtech.2009.03.006 . 19481826

[5] Turner P, Mamo G, Karlsson EN. Potential and utilization of thermophiles and thermostable enzymes in biorefining. Microb Cell Fact. 2007;6:9 Epub 2007/03/16. 1475-2859-6-9 [pii] 10.1186/1475-2859-6-9 17359551 PMC1851020

[6] Yeoman CJ, Han Y, Dodd D, Schroeder CM, Mackie RI, Cann IK. Thermostable enzymes as biocatalysts in the biofuel industry. Adv Appl Microbiol. 2010;70:1–55. Epub 2010/04/03. 10.1016/S0065-2164(10)70001-0 S0065-2164(10)70001-0 [pii]. . 20359453 PMC4561533

[7] Ng IS, Li CW, Yeh YF, Chen PT, Chir JL, Ma CH, et al A novel endo-glucanase from the thermophilic bacterium *Geobacillus* sp. 70PC53 with high activity and stability over a broad range of temperatures. Extremophiles. 2009;13(3):425–35. 10.1007/s00792-009-0228-4 . 19296197

[8] Rastogi G, Bhalla A, Adhikari A, Bischoff KM, Hughes SR, Christopher LP, et al Characterization of thermostable cellulases produced by *Bacillus* and *Geobacillus* strains. Bioresour Technol. 2010;101(22):8798–806. Epub 2010/07/06. 10.1016/j.biortech.2010.06.001 S0960-8524(10)00959-4 [pii]. . 20599378

[9] Zambare VP, Bhalla A, Muthukumarappan K, Sani RK, Christopher LP. Bioprocessing of agricultural residues to ethanol utilizing a cellulolytic extremophile. Extremophiles. 2011;15(5):611–8. 10.1007/s00792-011-0391-2 . 21837419

[10] Meng F, Ma L, Ji S, Yang W, Cao B. Isolation and characterization of *Bacillus subtilis* strain BY-3, a thermophilic and efficient cellulase-producing bacterium on untreated plant biomass. Lett Appl Microbiol. 2014;59(3):306–12. Epub 2014/04/30. 10.1111/lam.12276 . 24773580

[11] Santos CR, Paiva JH, Sforca ML, Neves JL, Navarro RZ, Cota J, et al Dissecting structure-function-stability relationships of a thermostable GH5-CBM3 cellulase from *Bacillus subtilis* 168. Biochem J. 2012;441(1):95–104. Epub 2011/09/02. 10.1042/BJ20110869 BJ20110869 [pii]. . 21880019

[12] Smith MA, Romero PA, Wu T, Brustad EM, Arnold FH. Chimeragenesis of distantly-related proteins by noncontiguous recombination. Protein Sci. 2013;22(2):231–8. Epub 2012/12/12. 10.1002/pro.2202 23225662 PMC3588919

[13] Smith MA, Rentmeister A, Snow CD, Wu T, Farrow MF, Mingardon F, et al A diverse set of family 48 bacterial glycoside hydrolase cellulases created by structure-guided recombination. FEBS J. 2012;279(24):4453–65. Epub 2012/10/19. 10.1111/febs.12032 . 23075376

[14] Heinzelman P, Snow CD, Smith MA, Yu X, Kannan A, Boulware K, et al SCHEMA recombination of a fungal cellulase uncovers a single mutation that contributes markedly to stability. J Biol Chem. 2009;284(39):26229–33. Epub 2009/07/25. 10.1074/jbc.C109.034058 C109.034058 [pii]. 19625252 PMC2785310

[15] Komor RS, Romero PA, Xie CB, Arnold FH. Highly thermostable fungal cellobiohydrolase I (Cel7A) engineered using predictive methods. Protein Eng Des Sel. 2012;25(12):827–33. Epub 2012/09/11. 10.1093/protein/gzs058 gzs058 [pii]. . 22961332

[16] Trudeau DL, Smith MA, Arnold FH. Innovation by homologous recombination. Curr Opin Chem Biol. 2013;17(6):902–9. 10.1016/j.cbpa.2013.10.007 . 24182747

[17] Li W, Zhang WW, Yang MM, Chen YL. Cloning of the thermostable cellulase gene from newly isolated *Bacillus subtilis* and its expression in *Escherichia coli*. Mol Biotechnol. 2008;40(2):195–201. Epub 2008/06/26. 10.1007/s12033-008-9079-y . 18576142

[18] Yang D, Weng H, Wang M, Xu W, Li Y, Yang H. Cloning and expression of a novel thermostable cellulase from newly isolated *Bacillus subtilis* strain I15. Mol Biol Rep. 2010;37(4):1923–9. Epub 2009/08/12. 10.1007/s11033-009-9635-y . 19669599

[19] Lee CC, Maestre-Reyna M, Hsu KC, Wang HC, Liu CI, Jeng WY, et al Crowning proteins: modulating the protein surface properties using crown ethers. Angew Chem Int Ed Engl. 2014;53(48):13054–8. Epub 2014/10/08. 10.1002/anie.201405664 25287606 PMC4288931

[20] Voigt CA, Martinez C, Wang ZG, Mayo SL, Arnold FH. Protein building blocks preserved by recombination. Nat Struct Biol. 2002;9(7):553–8. Epub 2002/06/04. 10.1038/nsb805 nsb805 [pii]. . 12042875

[21] Karypis G, Aggarwal R, Kumar V, Shashi S. Multilevel hypergraph partitioning: applications in VLSI domain. Very Large Scale Integration (VLSI) Systems, IEEE Transactions on. 1999;7(1):69–79. 10.1109/92.748202

[22] Karypis G, Kumar V. Multilevel k-way hypergraph partitioning. Vlsi Des. 2000;11(3):285–300. 10.1155/2000/19436 WOS:000165157300007.

[23] Zhang YH, Cui J, Lynd LR, Kuang LR. A transition from cellulose swelling to cellulose dissolution by o-phosphoric acid: evidence from enzymatic hydrolysis and supramolecular structure. Biomacromolecules. 2006;7(2):644–8. 10.1021/bm050799c . 16471942

[24] Sengupta S, Jana ML, Sengupta D, Naskar AK. A note on the estimation of microbial glycosidase activities by dinitrosalicylic acid reagent. Appl Microbiol Biotechnol. 2000;53(6):732–5. Epub 2000/08/05. . 10919335 10.1007/s002530000327

[25] Minor W, Tomchick D, Otwinowski Z. Strategies for macromolecular synchrotron crystallography. Structure. 2000;8(5):R105–10. Epub 2000/05/10. S0969212600001398 [pii]. 10801499. 10801499 10.1016/s0969-2126(00)00139-8

[26] Emsley P, Cowtan K. Coot: model-building tools for molecular graphics. Acta Crystallogr D Biol Crystallogr. 2004;60(Pt 12 Pt 1):2126–32. 10.1107/S0907444904019158 . 15572765

[27] Murshudov GN, Vagin AA, Dodson EJ. Refinement of macromolecular structures by the maximum-likelihood method. Acta Crystallogr D Biol Crystallogr. 1997;53(Pt 3):240–55. 10.1107/S0907444996012255 . 15299926

[28] Pettersen EF, Goddard TD, Huang CC, Couch GS, Greenblatt DM, Meng EC, et al UCSF Chimera—a visualization system for exploratory research and analysis. J Comput Chem. 2004;25(13):1605–12. 10.1002/jcc.20084 . 15264254

[29] Romero PA, Stone E, Lamb C, Chantranupong L, Krause A, Miklos AE, et al SCHEMA-designed variants of human Arginase I and II reveal sequence elements important to stability and catalysis. ACS Synth Biol. 2012;1(6):221–8. Epub 2012/06/28. 10.1021/sb300014t 22737599 PMC3378063

[30] Smith MA, Bedbrook CN, Wu T, Arnold FH. *Hypocrea jecorina* Cellobiohydrolase I Stabilizing Mutations Identified Using Noncontiguous Recombination. ACS Synth Biol. 2013 Epub 2013/05/22. 10.1021/sb400010m . 23688124

[31] Heinzelman P, Romero PA, Arnold FH. Efficient sampling of SCHEMA chimera families to identify useful sequence elements. Methods Enzymol. 2013;523:351–68. Epub 2013/02/21. 10.1016/B978-0-12-394292-0.00016-3 B978-0-12-394292-0.00016–3 [pii]. . 23422438

[32] Trudeau DL, Lee TM, Arnold FH. Engineered thermostable fungal cellulases exhibit efficient synergistic cellulose hydrolysis at elevated temperatures. Biotechnol Bioeng. 2014;111(12):2390–7. 10.1002/bit.25308 . 24916885

[33] Wu I, Arnold FH. Engineered thermostable fungal Cel6A and Cel7A cellobiohydrolases hydrolyze cellulose efficiently at elevated temperatures. Biotechnol Bioeng. 2013;110(7):1874–83. Epub 2013/02/14. 10.1002/bit.24864 . 23404363

[34] Zhang X-Z, Sathitsuksanoh N, Zhu Z, Percival Zhang YH. One-step production of lactate from cellulose as the sole carbon source without any other organic nutrient by recombinant cellulolytic Bacillus subtilis. Metabolic Engineering. 2011;13(4):364–72. 10.1016/j.ymben.2011.04.003 21549854

