# Supplementary material for: Exploring the Mechanism Responsible for Cellulase Thermostability by Structure-Guided Recombination
Source: PLoS One. 2016 Mar 17;11(3):e0147485. doi: 10.1371/journal.pone.0147485 (PMC4795783; doi:10.1371/journal.pone.0147485)
Supplement: S1 Table — (DOCX) [file pone.0147485.s001.docx]

**S1 Table. Data collection and refinement statistics^a^**

|  | GsCelA P1 | C10/18-crown-6 |
| --- | --- | --- |
| Data collection | | |
| Wavelength (Å) | 1.5418 | 1.000 |
| Space group | *P*2_1_2_1_2_1_ | *C*222_1_ |
| Cell dimensions (Å) | *a* =40.96, *b* = 71.67, *c* =92.25 | *a* =63.25, *b* = 83.25, *c* =121.60 |
| Resolution (Å) | 30.0-1.62 (1.68-1.62) | 25.0-1.50 (1.55-1.50) |
| Unique reflections | 35,214 | 51,327 |
| *R*_merge_ (%) | 7.4 (49.5) | 6.3 (33.4) |
| *I*/σ(*I*) | 32.4 (4.0) | 32.5 (5.1) |
| Completeness | 99.8 (98.3) | 99.5 (96.3) |
| Redundancy | 7.0 (5.8) | 7.7 (5.2) |
| Refinement | | |
| Resolution (Å) | 30-1.62 | 25-1.50 |
| No. of reflections *R*_work_/*R*_free_ | 33,088/1,760 | 43,576/2,478 |
| *R*_work_/*R*_free_ | 15.9/17.8 | 16.9/20.0 |
| No. of atoms/Avg B factor (Å^2^) | | |
| Protein | 2,513/10.9 | 2,430/14.9 |
| Water | 398/25.9 | 442/29.4 |
| Ca |  | 1/9.7 |
| 18-crown-6 |  | 36/21.7 |
| RMSD | | |
| Bond lengths (Å) | 0.007 | 0.009 |
| Bond angles (°) | 1.30 | 1.34 |
| Ramachandran statistics (%)^b^ | | |
| Most favored | 88.6 | 87.5 |
| Additionally allowed | 11.0 | 12.1 |
| Generously allowed | 0.4 | 0.4 |
| Disallowed | 0.0 | 0.0 |

^a^ Values corresponding to the highest resolution shell are shown in parentheses.

^b^ The stereochemistry of the model was validated with PROCHECK.
